# How vision and leadership shaped the U.S. National Cancer Institute's 50-year journey to advance the evidence base of cancer control and cancer care delivery research

**DOI:** 10.1016/j.hpopen.2020.100015

**Published:** 2020-10-13

**Authors:** Arnold D. Kaluzny, Donna M. O'Brien

**Affiliations:** a Gillings School of Global Public Health, Cecil G. Sheps Center for Health Services Research, Lineberger Comprehensive Cancer Center, University of North Carolina at Chapel Hill, Chapel Hill, NC, United States of America; b Strategic Visions in Healthcare LLC, New York, NY, United States of America; c International Cancer Expert Corps, Washington, DC, United States of America

**Keywords:** 1971 National Cancer Act, Cancer Control Policy to Practice, Cancer care delivery research, Cancer Care Continuum

## Abstract

In 1971, Congress passed the National Cancer Act, landmark legislation that reorganized the National Institutes of Health's National Cancer Institute (NCI). The Act included a new focus on cancer control, including the requirement that the NCI award research grants and contracts, in collaboration with other public agencies and private industry, to conduct cancer control activities related to the diagnosis, prevention, and treatment of cancer. The requirement placed the NCI at the nexus of a rapidly changing science and a complex and dynamic healthcare delivery system and involved an evolutionary transformation to advance cancer control and cancer care delivery research along the cancer care continuum. Analysis is based on a qualitative ethnographic approach using historical records, oral histories, and targeted interviews. The multimethod approach provided the opportunity to describe the vision, leadership, and struggle to build an infrastructure, expand expertise, and forge collaboration with the NCI and a complex and changing healthcare system. As the 50th anniversary of the National Cancer Act approaches in 2021, the process and these achievements are at risk of being taken for granted or lost in the flow of history. Documenting the process, milestones, and key players provides insight and guidance for continuing to improve cancer care, advance research, and reduce cancer incidence and mortality. Cancer care is a microcosm of the larger healthcare system providing insight and lessons on the importance of developing and maintaining a research infrastructure and the role of multi-level collaboration and partnerships involving both the private and public sectors.

## Highlights

• Fifty years ago the U.S. National Cancer Act mandated Cancer Control activities.
• Vision and leadership at the NCI were critical to advance cancer control on a global scale.
• Cancer care is a microcosm of challenges facing health policy globally.
• Evidence based strategies and infrastructure are important building blocks.
• Public-private collaboration is essential for meeting future challenges.

## 1 Introduction

In 2010, in his Pulitzer Prize-winning “biography” of the disease, Siddhartha Mukherjee [1] described cancer as the “emperor of all maladies.” Almost a decade later, the description is still apt. In 2018, in the U.S. alone, cancer claimed nearly 610,000 lives, and 1.7 million people were newly diagnosed with it. Human costs aside, the economic burden of cancer-related healthcare was $147.3 billion in 2017 [2]. That year, the World Health Assembly urged the promotion of cancer research “to improve the evidence base for cancer prevention and control” [3] – a concept pioneered in the United States with the 1971 passage of the National Cancer Act, often referred to as the “War on Cancer” [4,5].

The 1971 legislation led to expansion and reorganization of the NCI and required the NCI director to explore new opportunities to prevent cancer, diagnose it earlier, treat it more efficiently, and improve care and care outcomes [4]. The legislation empowered the NCI to leverage its unique role as a government-funded research institute in making long-term public investments to advance cancer care along the care continuum, employing innovative approaches and establishing relationships with the healthcare delivery system not accessible to the private sector [6].

Scientific discoveries were being made rapidly, and the NCI was called upon to translate the science for clinical application quickly and efficiently within a complex healthcare system. The science – and the complex healthcare system within which it was to be applied – was unimaginable in 1971.

For nearly 50 years, the U.S. National Cancer Institute (NCI) has worked to improve the evidence base of cancer prevention and control [5] and address the reality of a changing healthcare delivery system. While much progress has been made, there continue to be challenges. By 2013, the Institute of Medicine declared that the fragmented cancer care delivery system was “in crisis” [7] and called for new strategies to ensure that high-quality cancer care was offered. This paper is the story of the NCI's organizational evolution in cancer control as it navigated rapid changes in both science and in the healthcare delivery system. It is about vision, leadership and struggle to build an infrastructure, expand expertise, forge collaborations, and advance new ideas. Given the 50-year anniversary of the National Cancer Act in 2021, this evolution, including the achievements and the roles of individuals is important to document.

## 2 Methods and materials

The description of the expanding role of cancer control and the emergence of cancer care delivery research are based on a qualitative ethnographic analysis, using historical records, oral histories and targeted interviews, as well as observations by – and in some cases, the participation of – the authors as they were involved in policy decisions and program implementation during the 50-year study period. Historical records included a review of archived minutes of the NCI Board of Scientific Counselors, Board of Scientific Advisors (BSA) and relevant minutes of the NCI National Cancer Advisory Board (NCAB). Oral histories were commissioned and conducted in December 2008 and January 2009. The oral histories and written transcripts are archived in the Office of NIH History. In addition, key NCI personnel were interviewed in spring 2019.

## 3 Findings

### 3.1 The formative years

The 1971 legislation required the NCI to award research grants and conduct cancer control activities [4], but did not provide a mandate for funding. Lester Breslow, MD, MPH, director of the California State Health Department, and other public health leaders successfully advocated in 1973 to modify the National Cancer Act to include supplemental funding for cancer control [8].

To carry out the mandate effectively, it was essential that NCI develop new approaches to engage with the healthcare delivery system [9], even as that system and its providers were undergoing fundamental changes. Legislation that enacted Medicare and Medicaid was signed in 1965 [10], and the injection of funding to hospitals and physicians would dramatically change the relationship between hospitals and their management, and physicians [[11], [12], [13]].

#### 3.1.1 New leadership focus on cancer control and prevention

In 1981, Vincent DeVita, MD, director of the NCI's Division of Cancer Treatment, was appointed NCI director. A pioneer in the development of chemotherapy interventions, DeVita brought a dedication to empirical and protocol-based research [14]. He named Peter Greenwald, MD, DrPH, as director of NCI's Division of Resources, Centers and Community Activities (DRCCA). Greenwald, a physician with public health training, was well aware of the effects of a prevailing delivery system upon the implementation of cancer control programs. He had no history of working within NCI, but he and the NCI director shared a passion and respect for empirical research and a conviction that research must benefit society. In an interview, Greenwald recalled a conversation with DeVita:

“I wanted to do research that led directly to public benefit. To be successful, we needed to change the whole climate, the whole staff […] I felt that with Vince De Vita's backing, I would be able forcefully to change the nature of cancer control.”

[15]

Greenwald's appointment gave new perspective, power, and purpose to the concept of cancer control. Many of the programs did not align with his vision, and DRCCA did not include an identifiable cancer prevention focus. As Greenwald stated:

“Prevention was falsely defined as anything to do with studying causality, etiology, and epidemiology, with nothing that involved intervening to lower the occurrence of cancer. […] Research on causality is important, but it is not prevention.”

[15]

Greenwald assembled a group of like-minded physicians and researchers to design studies that could provide an empirical basis for prevention and control interventions. Within weeks of Greenwald's appointment, Joe Cullen, PhD, a behavioral scientist from the University of California at Los Angeles, was named the division's deputy director, and Jerome Yates, MD, a medical oncologist from Roswell Park Cancer Institute, was named chief of the Community Oncology and Rehabilitation Branch.

Cullen advocated for empirically-based programs that would make the most difference in cancer prevention. During his tenure, NCI enacted a new Smoking Tobacco and Cancer Prevention program to reduce tobacco use [16]. Yates's experience with community oncologists provided him with both a clinical perspective and awareness that physicians wanted access to NCI clinical trials.

The expanding NCI research enterprise called for greater access to patients for clinical trials. The Community Clinical Oncology Program (CCOP), launched in 1981, would effectively engage community oncologists in the NCI clinical trials program with accrual exceeding expectations [17,18]. Leslie Ford, MD, who recently had joined the branch, described the uniqueness and foresight of the program:

“This was pretty sweeping talk about community oncology to say that physicians practicing in their communities would actually do as well as cancer center and university physicians in terms of quality care.”

[19]

### 3.2 Finding direction

The 1983 name change of DRCCA to the Division of Cancer Prevention and Control (DCPC) supported Greenwald's vision of cancer control – as “a science based upon empirical research that leads to social benefit.” In 1984, Greenwald and Cullen published a paper that defined cancer control as a science involving the:

“… reduction of cancer incidence, morbidity and mortality through the orderly sequence from research interventions and their impact in a defined population to the broad,

systematic applications of the research results.”

[20]

The publication made a significant impact on the field. A transformation of the division's culture and operations acknowledged the interface between research and clinical practice, while taking into account the complex and changing healthcare system. Further changes were underway.

Ed Sondik, PhD, who was working at the National Heart, Lung, and Blood Institute, learned that Greenwald was considering a health services research branch to incorporate aspects of economics, operations research, and biometrics. Sondik recently had returned from Stanford University, where he conducted research on medical decision-making under uncertainty – a central focus of operations research [21].

In an interview with Greenwald, Sondik recalled:

“Whoever is talking about operations research at NIH is my kind of person […] because there is no activity like that at NIH […] There are of course the usual analytical sciences, epidemiology, demography, etc. … [but] … operations research is focused on decision making […] and that is quite crucial to health policy.”

[22]

Sondik was appointed head of the new Applied Research Branch (ARB) and focused on three areas of research – health services and economics, modeling and statistical methods, and cancer risk assessment.

An immediate product of this new branch was the publication of “Cancer Control Objectives for the Nation 1985–2000” [23]. The report targeted tobacco use, dietary factors, occupational hazards, and other cancer causes to reduce cancer deaths by as much as 50%. Unfortunately, the effort to implement the report was insufficient to have a major impact on smoking rates [24], and tobacco control research would remain an ongoing focus at the NCI. Still, the report and the new definition of cancer control were catalysts that led to several new research initiatives and productive collaborations between the NCI and state, local and federal governments, corporate leaders, and private organizations.

The national network of the CCOP was expanded in 1987 to include clinical trials for cancer prevention and control and to bring research to underserved populations [24,25]. The CCOP's expansion improved clinical practice [26] and was a model for other research networks [27]. Other evidence-based research efforts quickly followed.

The ambitious Prostate, Lung, Colorectal, and Ovarian (PLCO) Screening Trial [28], launched in 1991, would demonstrate, after 15 years, that screening had no significant effect on prostate, lung or ovarian mortality. For colorectal screening, there was a 21% reduction in incidence and a 26% reduction in mortality. This and other longitudinal colon cancer screening studies have led to public awareness campaigns, reimbursement changes, and enhanced advocacy efforts [29]. The Breast Cancer Surveillance Consortium (BCSC), established in 1994, collected screening data on patients and mammograms to track the relationship of screening to stage of diagnosis, survival, and breast cancer mortality [30] and led to enhanced understanding of clinical practice patterns, such as overutilization of screening [31].

#### 3.2.1 Translating evidence into the reality of clinical practice

In the late 1980s, Samuel Broder, MD, a medical oncologist and AIDS researcher who valued empirical research, succeeded DeVita as director and continued support of the NCI cancer prevention and control research program [32]. Evidence-based studies were challenging well-established guidelines recommending yearly breast cancer screening. By 1992, evidence suggested that annual breast cancer screening for premenopausal women ages 40–49 had little or no effect on mortality and came with attendant harms [33]. The paper reporting these findings set off a chain of events. NCI began a formal review that ultimately led to the presentation of a report at a 1993 meeting of the Board of Scientific Counselors [34].

The interest generated by this topic and the public meeting led to live TV coverage with provider and advocacy groups presenting their perspectives on the risks and benefits of breast cancer screening. The highly controversial statement issued by the Board, in part, stated that:

“There is general consensus among experts that…To date, randomized clinical trials have not shown a statistically significant reduction in mortality for women under the age of 50.”

[35]

In the aftermath, NCI continued to invest in research to evaluate the efficacy of breast cancer screening, but deferred to established expert groups, such as the U.S. Preventive Services Task Force, to make formal recommendations on clinical practice guidelines. The risk-benefit statement is updated periodically.

The speed of advancing science, its clinical application, and the changing delivery system have amplified the importance of evidence-based recommendations, which are based not only on scientific evidence but also on an understanding of how care is delivered. How this balance is best achieved remains an ongoing challenge.

### 3.3 ‘Hard and new choices’

In 1995, leadership of the NCI was passed to Richard Klausner, MD, chief of the Cell Biology and Metabolism Branch of the National Institute of Child Health and Human Development, who had spent his professional career at NIH. He set into motion a series of committees to review NCI's major functions. Klausner wanted to close the gap between the “power and beauty of molecular approaches to biology and what happens clinically [36].” He noted that “hard and new choices” were required.

Klausner proposed a reorganization of the NCI that included establishment of two divisions – the Division of Cancer Prevention (DCP) and the Division of Cancer Control and Population Sciences (DCCPS) [37]. Peter Greenwald, former DCPC director, was appointed director of the DCP. The new division would be based on biological markers and the design of chemoprevention agents to reduce cancer risk. DCPC's seven functional research branches, which had produced significant, practice-changing studies in cancer prevention, screening, and public health [38], would move to the new DCP. Missing in the new division was expertise to relate scientific advances to clinical practice and organizational providers.

Greenwald remained committed to the premise that cancer prevention should maintain a link to the delivery system if social benefit were to be achieved, and created a matrix structure to correspond to major cancer disease sites. As he described:

“The basic structure was fine, but the matrix part did not work very well … the division lacked the ability to put resources directly into the matrix teams. The organ site and cancer prevention groups remain […] and matrix teams are created on an ad-hoc basis.”

[39]

Barbara Rimer, DrPH, a Duke University social behavioral scientist who recently had completed a term as NCAB chair, was named the first DCCPS director. The appointment of a behavioral scientist as director of a major NCI division, along with the division's name change, acknowledged that the social and behavioral sciences played an important role in understanding a complex and changing delivery system. Robert Hiatt, MD, PhD, an epidemiologist from the University of California at San Francisco and member of the Cancer Control Advisory Committee, was appointed deputy director.

Building on the work of the DCPC and the ARB, Rimer and Hiatt quickly expanded existing databases to monitor practice and utilization patterns and recruited researchers to study social-behavioral interventions and cancer care delivery and outcomes [40]. The Surveillance, Epidemiology, and End Results program was expanded through collaborations with other federal agencies for data linkages [5]. To provide a greater focus on the care continuum, the Office of Cancer Survivorship, recently established to study the unique needs of the growing number of cancer survivors and respond to the expanding advocacy community [41,42], was incorporated into the division [43]. New programs were launched to examine the changing structure and operations of the delivery system, including the Cancer Research Network [44]. Established in 1998 to support cancer control research within integrated healthcare delivery systems, the network became a model for NCI and NIH, incorporating integrated delivery systems into their research programs [45,46].

To study how patient and provider factors influenced outcomes, the division received funding in 2001 for the Cancer Care Outcomes Research and Surveillance Consortium (CanCORS) [47], which examines how cancer patients' and providers' characteristics, beliefs, and behaviors influence treatment and outcomes. The program has contributed important findings that inform quality of life and symptom management issues for people with lung and colon cancer, including the need to implement supportive care strategies beginning at diagnosis [48].

“We knew we needed much bigger numbers and a more diverse base of participants to support research studies,” said Robert Croyle, PhD [49]. Croyle had served in the division as associate director of behavioral research, and in 2003, succeeded Rimer as director.

Increasing attention was given to understanding the patient's perspective and the measurement of patient-reported outcomes. In 2004, the division developed the Patient-Reported Outcomes Measurement Information System (PROMIS), an outcomes measurement now used internationally and applied to a wide variety of patient populations [50].

The need to assess and improve cancer screening practices and outcomes in a real-world setting led to the launch in 2011 of the multi-site Population-based Research to Optimize the Screening Process (PROSPR) program, which established an infrastructure and common metrics to study screening across disparate locations for breast, cervical, colorectal, and lung cancer. The program identified the need for greater understanding of factors that drive variation and of ways that new screening technologies and healthcare environment changes in policies and reimbursement were affecting screening. Its re-funding for five years in 2018 will support more in-depth study of measures for health system-level factors that impact the screening processes, including those that influence access and disparities, and will examine ways to ensure the quality of screening [51].

Recognizing the need for multilevel interventions [52], the division convened a forum to identify needed research, understand the current state of the science, and clarify issues in the conceptualization of this research across scientific disciplines [53]. In 2014, the division was reorganized into four research areas [5] that enable it to continue its unique role in funding the conduct of longitudinal studies with large patient populations, respond to changes in science, and understand the multilevel influence of an increasingly complex delivery system.

### 3.4 Building bridges

In 2001, the newly appointed NCI director brought increased attention to the delivery of cancer care and aimed to improve cancer outcomes. Andrew von Eschenbach, MD, a urologic oncologist and cancer center executive, had built his career at MD Anderson Cancer Center in Houston. As he described:

“Moving to the NCI was like going from boots on the ground, during which I was on the front line every day, involved with day-to-day cancer care, to being a pilot in an AWAC surveillance plane, where I get to see the whole landscape of oncology.”

[54]

Based on his clinical experience and now with a new “bird's eye” perspective [54], von Eschenbach set ambitious goals, with emphasis on the rapid acceleration of the discovery–development–delivery cycle and the application of nanotechnology, genomics, proteomics, and bioinformatics as they affect the full continuum of cancer care [55].

In 2005, von Eschenbach recruited John Niederhuber, MD, a surgeon with experience in basic sciences, to be deputy director. Niederhuber, who had served as NCAB chair and as director of the University of Wisconsin Comprehensive Cancer Center, accepted the position on condition that he could develop a program to expand community hospitals' ability to provide state-of-the-art cancer care [56]. Shortly after Niederhuber's arrival, however, President George W. Bush appointed von Eschenbach to lead the U.S. Food and Drug Administration, and Niederhuber subsequently was appointed NCI director in 2006.

#### 3.4.1 Science at a crossroads

Cancer research was at a crossroads. Large clinical trials required significant resources, while new funds were critical to advancing basic science and technologies associated with the sequencing of the genome, an event which, by all measures, represented a paradigm shift in cancer research [57]. NCI was operating under considerable financial constraints, and Niederhuber was forced to make difficult choices.

At that time, the NCI had approved a large prospective clinical study (STELLAR), estimated to cost between $50 million and $100 million, to culminate a 20-year research program evaluating chemotherapy agents for breast cancer prevention. Despite its having been approved in an extensive review process, with the unlikely prospect that a pharmaceutical company would undertake such a project given the limited return on investment, the director appointed an ad-hoc panel to reconsider the project [58]. At the June 14, 2006, meeting of the NCAB, the panel reported that it could not “offer strong endorsement of the trial as it was presented for funding [59].” While scientists on both sides were critical of the way in which the matter was handled [60], the decision was an inflection point for the NCI, in which the well-established standards of clinical trials were suspended to accommodate a rapidly evolving science [61].

#### 3.4.2 Partnering with the healthcare delivery system

In 2007, Niederhuber, based on his commitment to expand the capacity of community hospitals to provide state-of-the-art cancer care and growing interest in “precision oncology,” initiated the NCI Community Cancer Centers Program (NCCCP) pilot, a public-private partnership with 16 community hospitals [62], later expanded to 30 hospitals. The program was met with mixed reviews when presented to the NCAB and the Board of Scientific Advisors. Some felt the concept was “comprehensive and ambitious” and “addressed major healthcare issues of the time.” Others were skeptical that community hospitals would make the matching investment or wondered how the NCCCP was different from the well-established CCOP [55].

NCCCP was based in the Office of the NCI director, as its scope cut across several NCI divisions and centers. Unlike other NCI programs, it required the direct involvement of hospital management, as management controlled resources. Program oversight was managed by committee, with representatives from each hospital and from participating NCI divisions and centers. The result was a learning collaborative that facilitated rapid development and dissemination of strategies to achieve program goals [55].

An evaluation showed that program goals and co-investment requirements were met or exceeded [[63], [64], [65]]. Organizational factors associated with improved outcomes included the direct involvement of executive management, strengthened alignment between hospitals and their cancer specialty physicians, development of collaborative learning among participating hospitals, and access to NCI expertise for benchmarking and sharing best practices [66].

#### 3.4.3 Aligning NCI research programs to strengthen the relationship with the delivery system

In 2010, Harold Varmus, MD, succeeded Niederhuber as NCI director [67]. A Nobel laureate, former NIH director, and president of Memorial Sloan Kettering Cancer Center, Varmus quickly moved to emphasize a basic science agenda within NCI, prioritizing research project grants for investigator-initiated biomedical research. He named Greenwald associate director for cancer prevention in the Office of the NCI Director and appointed Barnett Kramer, MD, MPH, director of DCP. Kramer recently had retired as director of the NIH Office of Disease Prevention.

In a time of limited resources and increased investment in basic sciences, NCI in 2012 made the decision to merge NCCCP and CCOP, with Douglas Lowy, MD, deputy director of the NCI [68], facilitating this planning process. The new program, the NCI Community Oncology Research Program (NCORP), would be based within DCP, but would have an associate director from the DCCPS. As Kramer noted:

“Collaboration is the key. Building on our past collaboration with DCCPS, now more formalized through NCORP, DCP has access to broader expertise in health services and access to the delivery system to advance prevention and control research.”

[69]

#### 3.4.4 Cancer care delivery research (CCDR)

Researchers from DCCPS and DCP worked together to document their understanding of the healthcare system's structure, processes, and role in cancer care and research. For purposes of clarifying the NCORP research agenda, the group defined cancer care delivery research as:

“the multidisciplinary field of scientific investigation that studies how social factors, financing systems, organizational structures and processes, health technologies, and healthcare provider and patient behaviors affect access to cancer care, the quality and cost of cancer care, and ultimately the health and well-being of cancer patients and survivors.”

[70]

Launched in 2014, NCORP has been effective in clinical trial accrual and in its unique role as a community-based laboratory to advance the evidence base of cancer prevention and control, including the conduct of complex delivery-system-based studies for cancer control that can inform health policy and value-based care [71,72]. Based upon an external review, NCORP was approved for funding with a new six-year award made in 2019 [73,74].

## 4 Discussion

Today's pressing issues in prevention and control little resemble those identified in NCI's formative years [7,75,76]. Cancer prevention and control research in the U.S. now incorporates the multi-level complexity [77,78] of the financing and delivery of cancer care, focusing upon rescinding ineffective, low-value, or harmful practices [[79], [80], [81]]; continuing tobacco control efforts [82]; better engagement of patients in decision making and meeting the needs of “cancer survivors” [[83], [84], [85]]; reducing disparities [86,87]; and assessing new reimbursement models for value-based care [[88], [89], [90]]. These issues suggest a “new frontier”– one requiring new methods and access to large datasets and analytical capacity as advanced by Norman "Ned" Sharpless, MD who was appointed Director of the NCI in 2017 [91].

In 2016, the U.S. Cancer Moonshot initiative was launched [92], and Congress passed the 21st Century Cures Act [93]. These initiatives, which provide $1.8 billion over seven years, expand research opportunities, including the basic concept of cancer control, precision prevention and early detection, expansion of clinical trials, enhanced data sharing, and implementation sciences. The complexity of advancing the science and improving the evidence base of cancer prevention and control through greater collaboration among various federal agencies was documented in a 2019 report issued by the U.S. National Academies of Sciences, Engineering and Medicine (NASEM) [94].

For the future, to optimize that value of cancer control and cancer care delivery research at the NCI it will be important to:

• **Maintain a strong infrastructure**. The public sector and the NIH/NCI have played an important role in many clinical practice advances that are now taken for granted. With funding from Congress, the NCI has provided the infrastructure and served as a catalyst for advances along the cancer continuum. More than ever, these efforts are needed to meet the challenges of an advancing science, clinical application and a complex and evolving healthcare system.
  The value of this infrastructure became evident with the COVID-19 pandemic, as the NCI rapidly mobilized on many levels [95]. Patients on clinical trials became an urgent priority with NCI, and its investigators quickly “re-imagined” ways to manage the care of these patients so their treatment was not compromised. They also recognized that some lessons learned may carry forward as new best practices. With cancer patients at particular risk, cancer control and cancer care delivery studies were immediately established and made available through the NCORP and other clinical trial programs. The NCI COVID-19 Cancer Patient Study (NCCAPS), a large cohort natural history study, is tracking how the disease develops and changes in patients undergoing treatment for cancer and the immediate and long-term effects [96]. Maintaining an infrastructure to be ready for conducting studies such as this is essential for making progress.
• **Expand on partnerships.** The NASEM report called for coordination of cancer control efforts across various federal agencies so that relevant issues, such as quality, scientific advances, safety, and cost and payment, could be addressed in an integrated way across the sectors involved in the delivery of care. Actions and funding to facilitate these partnerships are needed.
  Partnering with providers is also critical. Programs such as the NCORP, with its national network of community oncologists and healthcare organizations and systems, offer the capacity to collaborate with the clinical community to develop evidence-based interventions across the full continuum of care. Such interventions include evaluation to improve care processes, assess alternative reimbursement models, and study new care delivery models as changes in science and the health system accelerate at an unprecedented rate. Finding ways to expedite the timeframe for the study of these urgent issues, as has happened for COVID-19, will be important for leveraging the value of these programs.

These efforts built upon the 1971 National Cancer Act, and after nearly a half-century, the NCI, in its historic and catalytic role as a government-funded research institute, has been joined in its efforts by other public [97] and private-sector organizations [98] and professional associations [99] that contribute to progress. Much remains to be done. Yet the expanding evidence base of cancer prevention and control and the integration of cutting-edge science with public and private-sector vision and leadership have transformed cancer care and the lives of those facing cancer. The challenge moving forward is to leverage what has been accomplished, in collaboration with efforts in the public and private sectors, and more fully to engage those in the healthcare system as partners in research along the full care continuum – from risk assessment and prevention through survivorship and end of life [100].

## 5 Conclusions

When the 1971 National Cancer Act was passed, the language of a “war on cancer,” with the implication of being able to *win* that war, was used to mobilize support for consequential legislation that has led to new knowledge, prevention and better outcomes for patients. However, the complexity of the disease – and the challenges of making excellent cancer care more universal through both basic and cancer control research – made it clear that transformation would not be immediate. Six years later, Benno Schmidt Sr., a key player in the passage of the National Cancer Act and chair of the first President's Cancer Panel (PCP), would note in his 1977 PCP report that the national cancer program was a vast undertaking requiring patience and constancy of support by Congress, the federal administration, and the public.

Moving forward, continuing advances in science and clinical application within a changing healthcare system will present unrelenting challenges to the provision of high-quality health and cancer care. Though the challenges are significant, the NCI, with its committed leadership, expertise and infrastructure, and with increased efforts to forge partnerships, will continue to play a central and catalytic role in expanding the evidence base of cancer control and cancer care delivery research. Despite this half-century of phenomenal progress, the complexity of cancer remains and calls for continued study of its implications for the continuum of cancer care and the changing healthcare system. “The goal,” as expressed by Schmidt decades ago and still true today, “is the course we travel together, and the end is only the beginning.” [101].

## CRediT authorship contribution statement

ADK and DOB were involved in the conceptualization, research, interviews, document review and the writing and editing of the manuscript.
